# Quantification of pesticide dosage and determinants of excessive pesticide use in smallholder vegetable production systems in Tanzania

**DOI:** 10.1016/j.heliyon.2024.e41070

**Published:** 2024-12-07

**Authors:** Jones A. Kapeleka

**Affiliations:** Tanzania Plant Health and Pesticides Authority (TPHPA), P.O. Box 3024, Arusha, Tanzania

**Keywords:** Pesticides active ingredient, Pesticides exposure, Insecticides, Fungicides, Insect pests

## Abstract

The use of pesticides for diseases and insect pest control has become a key component in smallholder vegetable production. This study therefore quantified the concentration of pesticide active ingredient per unit production land (kg a.i/ha), and drivers of increased pesticide use in smallholder vegetable production systems in Tanzania. Through field surveys and observations, data were collected from 385 farmers from Iringa, Arusha, and Kilimanjaro regions. A binary probit model was used to derive factors fostering increased pesticide use. High dose rates with increased frequency of application were evident. The concentrations of active ingredients (kg a.i/ha) were far above the world averages for fungicides (17.18 kg a.i/ha in tomato and 13.05 kg a.i/ha in onion), insecticides (5.86 kg a.i/ha in tomato and 6.37 kg a.i/ha in onions) and herbicides (3.78 kg a.i/ha in onions). Furthermore, 47.9 % of all pesticides were wrongly used. Most farmers (88.6 %) lacked knowledge of pest control and 88.9 % of farmers were unaware of pesticide safety practices. There was an increasing trend in pesticide use (58.4 %). WHO Class II hazard-classified pesticides (68.9 %) dominated smallholder vegetable production. Extremely hazardous (Class Ia) and highly hazardous (Class Ib) pesticides were also used. The binary probit model showed that the number of crops grown, pesticide mixing, and region contributed positively to the likelihood of increased pesticide use. In contrast, farmers’ perception of the effectiveness of pesticides, lack of access to safe use information, poor use of safety gear, and inability to read pesticide labels had a negative impact. Excessive pesticide use jeopardizes the sustainability of smallholder vegetable production. Pesticide control and monitoring at the farm level, restricted use of highly hazardous pesticides, application of greener pesticides, and the use of specialized pesticide spray men in smallholder vegetable production would lessen and protect farmers and the environment from pesticide exposure.

## 1 Introduction

In Tanzania, smallholder vegetable production is characterized by minimal productivity in small scattered plots, dominated by excessive pesticide use. According to The National Horticulture Development Strategy and Action Plan 2021–2031, horticulture is an important sub-sector, growing at 9 to 12 percent per annum while the agriculture sector as a whole grows at 4 %. It employs more than 4.5 million people with 65–70 % being women and youth and earning the country more than USD779 million per annum, making a significant contribution to food security, nutrition improvements, and economic growth. The horticultural industry is mainly practiced by small-scale farmers with a few large-scale operators. About 70 % of the export of fruits and vegetables depends on farmers with land holding less than 2 acres [1].

Due to the high demand for horticultural crops, production is year-round through the use of irrigation systems [2]. Smallholder vegetable farmers depend heavily on the use of pesticides for the control of different pests and diseases. This is probably because they believe that the only solution to pest problems is to spray more frequently and use different types of pesticides [3]. Pesticides are a diverse group of chemicals particularly created for the management of insect pests, weeds, and plant diseases. These substances contain special qualities intended to be poisonous to pests [4]. They are defined by Tanzania's Plant Health Act No. 4 (2020) as substances or chemicals used for preventing, eliminating, repelling, or neutralizing any kind of pest. These substances include insecticides, fungicides, herbicides, rodenticides, avicides, nematicides, antimicrobials, and molluscicides.

Thus, they have a significant contribution in preventing and controlling insect pests, diseases, weeds, and other plant pathogens to reduce or eliminate yield losses and get high-quality products [5,6]. The use of pesticides classified as highly hazardous by the World Health Organization (WHO) is common in many developing countries. Organophosphates, carbamates, and synthetic pyrethroids are the most common pesticides in Tanzania like other developing countries. Banned or restricted pesticides in many developed countries have been reported to be used in developing countries [3,7,8]. Nonetheless, these chemicals have the potential to cause adverse effects on human and environmental health [[9], [10], [11], [12]].

Poor knowledge of the use and handling of pesticides among farmers has been reported in different studies [3,8,[13], [14], [15], [16], [17], [18], [19], [20]]. Pesticide misuse therefore continues to be a major problem in horticultural farming. Pesticide containers being thrown in the environment after usage, farmers' unawareness of the existence of residues after application, and mixing of different pesticides with increased dosage and high frequency of application [13,15,16,[21], [22], [23]] had been common malpractices of pesticide use. In Tanzania, the main issues reported include farmers’ knowledge, pesticide poisoning, practices, and perceptions on pesticide use, pesticide exposure and associated health risks, pesticide use and handling practices, risk behaviors, vulnerable groups, biomarkers of exposure, environmental exposure, and disposal of empty pesticides containers [2,12,15,16,18,24,25]. Poor extension services, farmers not being informed on judicious pesticides, education, and awareness are often reported as major drawbacks to effective pesticide use [12,16,[20], [21], [22],[25], [26], [27], [28], [29], [30], [31]]. Nonetheless, most organizational and governmental programs encourage the use of pesticides focusing on increasing production yield with little or no emphasis on their health and environmental risks and consequences [32].

The need for a paradigm shift in farmers' and government views of policies on pesticides and their handling has led to numerous studies being conducted [3,8,[13], [14], [15], [16], [17], [18], [19], [20]], yet there is no or little change in pesticide handling practices among farmers. Though there have been several studies describing scenarios of pesticide use and misuse [7,17,19,20,23,26,27,33], information on the dynamics of pesticide uses, pesticide dosage, application rates, and volumes of pesticides are hardly documented in Tanzania. The study therefore significantly quantifies concentrations of active ingredients in spray mixtures and the corresponding application rates It provides useful information on active ingredients per unit cropping land, farmers’ behavior, and changing practices of pesticide use in smallholder vegetable production systems and associated human and environmental exposure risks.

## 2 Materials and methods

### 2.1 Study area

The survey was undertaken in the Southern Highlands (Iringa) and Northern Corridor (Arusha). The decision to choose these geographical areas was based on high vegetable productivity and extensive use of pesticides. Iringa region covers an area of 35,503 km^2^ and an average of 345,000 ha (3,4501 km^2^) of land used for agricultural production it is situated at a latitude of 7.77°S and longitude of 35.69°E. Arusha, located at latitude 3° 23′ S and longitude 36° 40’ E covers 37,576 km^2^ large part of which is covered by nature and wildlife conservation. Most smallholder farmers in these areas are involved in producing vegetable crops that are widely consumed in Tanzania, including tomatoes, onions, watermelons, and sweet peppers. Other leafy vegetables include Chinese cabbage, carrots, cucumbers, cabbage, amaranths, kale and nightshade.

### 2.2 Study design

A descriptive cross-sectional survey design was used in the assessment of pesticide usage to determine how frequently, widely, and increasingly are pesticides applied, the dosage in pesticide mixtures, management, storage, and disposal of pesticides. Data were collected during high spraying seasons from farmers with similar social economic variables and farming practices. This method is often used to make inferences about possible relationships. Likewise, it can be used to estimate the odd ratios to study the association between behaviors and outcomes [34].

### 2.3 Sampling method

Purposive sampling was employed in selecting regions and districts under study. This sampling technique enables the target sample with knowledge and information on the subject matter to be reached quickly [3]. After selecting the regions and districts, villages with intensive vegetable production were also purposely selected. A total of 20 villages, 4 from each district with 3218 vegetable farmer households were involved in the study (Table 1). In obtaining cross-sectional data, simple random sampling without replacement was used to select households to be included in the study. Randomness was achieved by assigning random numbers to the list of participants from which the sample was collected. Vegetable farmers were randomly selected from the list of households provided by respective village government officers in proportion to the total number of households involved in vegetable production. A sample of 385 farmers was used in this study. Of the 420 survey questionnaires that were administered, 385 were correctly filled, and hence the response rate of 92 %.

**Table 1:** Distribution of farmers and farm households.

Village of respondent	Number of farmers	%	Number of households	%
Kimashuku	35	9.1	298	9.3
Ngabobo	30	7.8	252	7.8
Mtitu-Manimbi	30	7.8	230	7.1
Ihimbo	25	6.5	218	6.8
Olkung'wado	25	6.5	205	6.4
Qangded	25	6.5	195	6.1
Mbuga Nyekundu	20	5.2	186	5.8
Uwiro	20	5.2	165	5.1
Barazani	20	5.2	201	6.2
Modio	20	5.2	183	5.7
Muhimbili	20	5.2	180	5.6
Sonu	18	4.7	134	4.2
Roo	15	3.9	122	3.8
Kiboyeye	15	3.9	118	3.7
Shirimatunda	15	3.9	115	3.6
Mtitu-Ngugi	12	3.1	97	3.0
Mtitu-Magharibi	10	2.6	85	2.6
Itengule	10	2.6	83	2.6
Image	10	2.6	77	2.4
Kambi ya Simba	10	2.6	74	2.3
Total	385	100.0	3218	100.0

### 2.4 Sample size

The sample size was calculated based on Equation (1) for the determination of minimum sample size. This equation is applicable when the total population of interest is unknown [35].

Equation (1) $$n=\frac{Z^{2}p\left(1-p\right)}{M^{2}}$$

Where n = Sample size, Z = % point of the standard normal distribution which is 1.96, in this case, corresponding to a 95 % confidence level, M = marginal error which is 5 %, p = expected proportion of the respondents taken as 50 %, = 0.5, q = 1-p.

### 2.5 Research hypothesis

(a) Based on previous studies, this study hypothesized that in reducing the effects of insect pests, farmers have resorted to using more pesticides.
(b) Age, sex, mixing practices, poor extension services, region of the farmers, acreage under cultivation, frequency of pesticide application, number of crops grown, and low knowledge of pesticide usage are attributed to overuse of different pesticides.

### 2.6 Data collection procedures

A semi-structured questionnaire containing 36 closed and open-ended questions was administered to participants during the survey. The questionnaire used in previous studies [17] was used with minor modifications to suit the current research objectives. This improved questionnaire was pretested in the study areas. The reliability of the questionnaire was measured by administering the improved questionnaire among 20 individuals twice over one month from one village in the study areas, which was finally removed from the sample.

Data were collected by trained interviewers through face-to-face interviews and responses were recorded during interviews. Interviewers were trained in the management of data collection tools before interviews. All questions were translated and administered in Kiswahili, an official language in Tanzania. Demographic information such as age, education, access to training in pesticide use, agricultural production, insect pests/disease situation Information on perception of the effectiveness of pesticides, the use of PPEs, frequency of pesticide application, types of crops grown and number of pesticides mixed in a single mixture was also collected as explanatory variables for the determinants of increased pesticides use. Furthermore, data on the amounts of pesticides, concentrations, and application rates used was collected.

### 2.7 Data processing and analysis

Data were analyzed using SPSS 18.0 version computer software. Descriptive statistics, namely frequencies, percentages, and means, as well as cross-tabulation were used to summarize information among different variables. Quantitative data are presented as means and standard deviations (Mean ± SD). Pearson correlations were used to assess the relationship and significance of the association between increased levels of pesticide use and demographic variables. Chi-squared (χ^2^) test was used to compare frequencies and to determine significant differences in the proportions of responses given.

The quantity of pesticides applied per unit of cropping land was calculated as per Equation (2).

Equation 2 Dose Applied = Concentration (g a.i/L) x Rate (L/ha)

Where Dose Applied = Dose of pesticides applied (g a.i/ha), Concentration = Concentration of the active ingredient (g a.i/L) and Rate = Application rate (L/ha).

The levels of pesticides used were dichotomized into high and low at one standard deviation below the mean levels of pesticide dose rate sprayed per acre of cropping land. Determinants of farmers’ changing patterns of increased pesticide use were determined by regressing the levels of pesticide use (dependent variable) on a set of demographic and pesticide handling practices variables using a binary probit model. A significant level for the results was accepted at p < 0.05.

The regression model used to estimate the determinants of increased pesticide use (IPU) is as per Equation (3).

Equation 3 IPU= (β_0_ + β_1_GF + β_2_AF + β_3_EL + β_4_NC + β_5_FS + β_6_PE + β_7_MP + β_8_AI + β_9_FA + β_10_SP + β_11_PPE + β_12_PL + β_13_RF + Ԑ)

Where IPU= Increased pesticide use, GF= Gender of Farmer, AF=Age of Farmer, EL = Education Level, NC= Number of vegetable Crops each farmer grows, FS= Farm Size, PE= Perception of the Effectiveness of Pesticides, MP = Mixing Practices, AI= Access to Information on Pesticide Use, FA= Frequency of Pesticides Application, SP = Source of Pesticides, PPE= Use of Personal Protection Equipment, PL=Read Pesticide Label, RF= Region of the farmer, Ԑ = unknown parameters, βi is the vector of regression coefficients being estimated, such that, for a one-unit increase in the predictor, it is expected that the response variable level will change by its respective regression coefficient, other variables in the model being held constant.

The focus of the probit model is usually on the individual decision-making units [36]. The goal of the model is to calculate the likelihood that an observation with a given set of attributes will fall into a certain proposed category [8]. This model had been previously used to determine factors influencing knowledge of pesticide use, exploring farmers' knowledge of pesticide use and factors affecting application and pesticide use practices among vegetable farmers as well as explaining determinants of health impacts from pesticide use [3,8]. This binary probit model was therefore instrumental in predicting the likelihood that farmers' behavior will resort to increased use of pesticides based on specific predictors. The region was included in the model to ascertain possible differences in farmers’ level of pesticide use concerning their geographical location.

### 2.8 Ethical clearance

Ethical clearance was obtained from Tanzania's National Institute of Medical Research (NIMR) with Reference No. NIRM/HQ/R.8a/Vol.IX/2742. The respondents had the freedom to participate in the survey. Each participant signed a written consent form for participation in the research.

## 3 Results

### 3.1 Respondent's demographic information

The survey to assess pesticide use practices was done in three vegetable production regions namely Arusha (46.8 %), Iringa (29.1 %), and Kilimanjaro (24.2 %). A total of 385 farmers were interviewed from four districts; Kilolo (29.1 %), Arumeru (28.6 %), Hai (24.2 %), and 18.2 % Karatu, from which 20 villages were visited (Table 2), covering the two main vegetable production agro-ecological zones, namely, northern corridor and southern highlands.

**Table 2:** Demographic information from the study area.

Variables		n	%	p-values
Gender of respondent	Male	300	77.9	0.00
	Female	85	22.1	
**Total**		**385**	**100**	
The age category of respondent	15–24 years	15	3.9	0.00
	25–34 years	122	31.7	
	35–44 years	127	33	
	45–54 years	96	24.9	
	55–64 years	21	5.5	
	65 and above	4	1	
**Total**		**385**	**100**	
The highest education level attained	Never gone to school	24	6.3	0.00
	Primary education	305	79.4	
	Ordinary-level Secondary education	52	13.5	
	Advanced-level Secondary education	3	0.8	
**Total**		**384**	**100**	
Region of respondent	Arusha	180	46.8	0.00
	Iringa	112	29.1	
	Kilimanjaro	93	24.2	
**Total**		**385**	**100**	
District of respondent	Kilolo	112	29.1	0.00
	Arumeru	110	28.6	
	Hai	93	24.2	
	Karatu	70	18.2	
**Total**		**385**	**100**	

Males (77.9 %) dominated the smallholder vegetable production compared with females (22.1 %). The farming population was relatively younger. Youths (25–34 years) and middle-aged individuals (35–44 years) dominated smallholder vegetable production (31.7 % and 33.0 % respectively). The education level was generally low. Most farmers (79.4 %) had attained primary-level education, while only 13.5 % had attained ordinary-level secondary education (Table 2). Smallholder vegetable production is characterized by limited area under production. The average area under production per household was found to be 1.24 acres with no remarkable differences in all the regions surveyed (Data not shown).

### 3.2 Pesticides types and use practices

All farmers reported the use of pesticides. Insecticides (56.8 %), fungicides (39.2 %), and to a small extent, herbicides (1.4 %) were the main pesticides used. No alternative methods of pest control were reported. Misuse of pesticides in smallholder horticulture production was noted as well. Based on the list of registered pesticides, 52.1 % were correctly used for target crops, while 47.9 % were improperly used. These included banned pesticide products, unregistered pesticides, and pesticides registered for use in controlling different pests in other crops such as coffee, cashew nuts, and ornamental flower production.

Smallholder vegetable producers have limited access to extension services to advise them on insect pest control. Table 3 shows that 88.6 % had not received agricultural experts' advice on insect pest control in the last three years. Likewise, 88.9 % had not received any advice on the safe use of pesticides. Very few farmers (11.1 %) had received some training on the safe use of pesticides, mainly received from pesticide retailers (47.5 %), extension officers (45.0 %), and NGOs (7.5 %).

**Table 3:** Pesticides use and practices in vegetable production.

Variable			n	%	p-values
Categories of pesticides used in tomatoes		Insecticides	42	57	0.004
		Fungicides	29	39	
		Acaricides	2	3	
		Herbicides	1	1	
**Total**			**301**	**100**	
Farmers' use of pesticides		Correct use	49	52	0.000
		Wrong use	45	48	
**Total**			**301**	**100**	
Have received agricultural experts' advice on pest control		No	335	89	0.000
		Yes	43	11	
**Total**			**378**	**100**	
Have received experts' advice on pesticide-safe use		No	336	89	0.000
		Yes	42	11	
**Total**			**378**	**100**	
If yes, name the source.		Pesticide's sales agents	19	48	0.002
		Extension officer	18	45	
		NGO's	3	8	
**Total**			**40**	**100**	
State of current pesticide use		Had increased	213	58	0.001
		Had reduced	89	24	
		Remained virtually the same	60	16	
		I don't know	3	1	
**Total**			**365**	**100**	
Mix more than one pesticide during spraying		Yes	257	71	0.000
		No	104	29	
**Total**			**361**	**100**	
Number of pesticides in a pesticide cocktail	Four	114	47	0.003	
	Three	72	30		
	Five	27	11		
	Two	24	10		
	Six	6	3		
**Total**		**243**	**100**		

The rate of pesticide use has increased in the recent past (58.4 %). Farmers (71.2 %) mixed two or more pesticides in the mixing tank/backpack sprayer. The reasons for mixing pesticides included minimizing the spraying costs (32 %), increasing pesticide effectiveness (28.1 %), controlling all pests at once (26.1 %), and ensuring that the pesticides complement each other in controlling pests (13.8 %). Up to six different pesticides were mixed in a single tank. Close to half of the farmers (46.9 %) mixed four different pesticides in one mixing tank during spraying, 29.6 % mixed three pesticides, and 11.1 % mixed up to five different pesticides (Table 3).

### 3.3 Pesticide dose rate and volumes used in vegetable production

The most used mixing equipment were drums (200 L) and knapsack sprayers (20 L) (67.8 % and 32.8 % respectively). The high dose rate of pesticides was a common practice among smallholder vegetable producers. The mean active ingredient per hectare of production land for insecticides was 5.86 (kg a.i/ha) and 17.18 (kg a.i/ha) fungicides in tomatoes and 6.37 (kg a.i/ha) insecticides and 13.05 (kg a.i/ha) fungicides in onion and 3.78 kg a.i/ha of herbicides were applied in onion. An average of 3 spray men in both onion and tomato product spray for 5 working hours daily, indicating high pesticide contact hours and high-risk exposure due to tight spraying schedule (Table 4).

**Table 4:** Pesticide dose rates and volumes used in vegetable production.

	Minimum	Maximum	Mean
Spraying hours/day	1.00	12.00	5.06
Number of spray men	1.00	10.00	3.33
Tomato fungicide dose rate (kg a.i/ha)	12.54	21.80	17.18
Tomato insecticide dose rate (kg a.i/ha)	0.54	18.84	5.86
Onion fungicide dose rate (kg a.i/ha)	2.63	21.00	13.05
Onion insecticide dose rate (kg a.i/ha)	0.27	13.65	6.37
Onion herbicide dose rate (kg a.i/ha)	1.87	11.67	3.78

### 3.4 Frequency of pesticide use and management of empty pesticide containers

The main disposal method for empty pesticide containers was burning (58 %), burying (15 %), throwing in dustbins (12 %), and leaving empty containers in the fields (8 %) and very few farmers used empty pesticide containers for storing water and food (3 % and 2 %), respectively. Most farmers (70.3 %) did not read pesticide labels before using pesticides and 83.8 % did not follow the instructions. Most farmers (61 %) spray pesticides once a week, 18 % spray twice a week, and 12 % spray once in two weeks. This is a considerably high spraying frequency which translates into the spraying of an average of four times a month.

Most pesticides (78.5 %) used in smallholder vegetable production had full registration status, while (19.4 %) were not registered when checked against the list of registered pesticides. Furthermore, 68.9 % of all reported pesticides fell under Class II (Moderately Hazardous) of the WHO hazard classification of pesticides whereas 24.3 % were under Class U (Unlikely to present acute hazard in normal use). Small quantities of extremely hazardous (Class Ia) and highly hazardous (Class Ib) were also found to be used. About three-quarters (76.6 %) placed pesticides in a pesticide store while others stored them in their farms, general stores, and bathrooms/toilets (Table 5).

**Table 5:** Pesticides label use and management of empty pesticide containers.

Variable		n	%	p-values
Do you read the instructions on pesticide labels before use?	No	260	70	0.000
	Yes	81	22	
	Sometimes	29	8	
**Total**		**370**	**100**	
If yes, do you follow them?	No	227	84	0.000
	Yes	36	13	
	Sometimes	8	3	
**Total**		**271**	**100**	
What do you do with empty containers?	Burn them	212	58	0.000
	Buried	56	15	
	Thrown in the dustbin	42	12	
	Left in the field	30	8	
	Storing water	9	3	
	Store food	9	3	
	Thrown in a pit latrine	8	2	
**Total**		**366**	**100**	
Frequency of pesticide spraying	Once per week	224	61	0.000
	Twice per week	67	18	
	Once in two weeks	44	12	
	Three times per week	17	5	
	Twice a month	12	3	
	Once a month	4	1	
**Total**		**368**	**100**	
Registration status of pesticides used in Tomato	Full registration	73	79	0.001
	Not registered	18	19	
	Banned	1	1	
	Provisional registration	1	1	
**Total**		**93**	**100**	
WHO Classification of Pesticides used in Tomato	Class II (Moderately hazardous)	51	69	
	Class U (Unlikely to present acute hazard in normal use)	15	20	
	Not listed	4	5	0.000
	Class Ia (Extremely Hazardous)	1	1	
	Class Ib (Highly hazardous)	1	1	
**Total**		**74**	**100**	
				
Where do you store pesticides?	Pesticide's store	271	77	
	In the farm	27	8	0.000
	General store	26	7	
	Bathroom/toilet	14	4	
	Hang under the tree	7	2	
	Kitchen	6	2	
	Living room	3	1	
**Total**		**354**	**100**	

### 3.5 Types of pesticides used in smallholder vegetable production

Over 75 different pesticides were found to be used in smallholder vegetable production. Organophosphorus (29.3 %), Carbamates (16.2 %), and Substituted benzene (10.4 %) constitute the main chemical families of pesticides used. Others were a combination of Pyrethroid and Organophosphorus, Avermectin, a combination of Carbamate, Acylalanine, and Dithiocarbamate (Table 6).

**Table 6:** Pesticides chemical families used in smallholder vegetable production.

Variable		n	%
Chemical families of pesticides sprayed on tomatoes	Organophosphorus	285	29.3
	Carbamate	158	16.2
	Substituted benzene	101	10.4
	Pyrethroid + Organophosphorus	84	8.6
	Avermectin	82	8.4
	Carbamate + Acylalanine	67	6.9
	Dithiocarbamate	57	5.9
	Inorganic fungicide	39	4.0
	Pyrethroid	26	2.7
	Organochlorine	26	2.7
	Pyrethroid + Nitroimidazolidylideneamine	25	2.6
	Oxadiazines	12	1.2
	Conazole	6	0.6
	Propionic acid	5	0.5

### 3.6 Pesticides safety and use of personal protection equipment (PPE)

Most farmers did not have the basic and essential personal protective equipment for use during pesticide handling. Close to three-quarters (74.5 %) did not wear gloves when handling pesticides, 86.6 % did not use respirators, 82.6 % had no masks, 91.5 % did not wear goggles, and 79.3 % did not wear overalls when handling pesticides. Gumboots (60.3 %) were the most PPE used mostly in tomato fields, while farmers in onion fields were observed spraying barefooted (Table 7). Further investigation revealed that these PPE are often not available in retail stores where they purchase pesticides and are often sold at high prices whenever they are available.

**Table 7:** Personal protective equipment used by farmers.

Variable		n	%	p-values
Gloves	No	272	75	0.000
	I use and is in good order	73	20	
	Have but don't use	20	6	
**Total**		**365**	**100**	
Boots	I use and is in good order	219	60	0.000
	No	119	33	
	Have but won out	21	6	
	Have but don't use	4	1	
**Total**		**363**	**100**	
Respirator	No	309	87	0.000
	I use and is in good order	42	12	
	Have but don't use	6	2	
**Total**		**357**	**100**	
Mask	No	300	83	
	I use and is in good order	53	15	0.000
	Have but won out	8	2	
	Have but don't use	2	1	
**Total**		**363**	**100**	
Goggles	No	332	92	0.000
	I use and is in good order	21	6	
	Have but don't use	6	2	
	Have but won out	4	1	
**Total**		**363**	**100**	
Overalls	No	288	79	0.000
	I use and is in good order	66	18	
	Have but don't use	8	2	
	Have but won out	1	0	
**Total**		**363**	**100**	
Headcover	No	307	85	0.000
	I use and is in good order	48	13	
	Have but don't use	6	2	
**Total**		**361**	**100**	

### 3.7 Drivers of increased pesticide use

Generally, farmers used increased application rates with high pesticide volumes both in vegetable production systems. The correlation coefficients between increased pesticide use and demographic variables showed that increased pesticide use had a significant positive correlation with mixing more than one pesticide during spraying, the number of crops grown consecutively, and the region of the respondent. On the other hand, increased pesticide use had a significant negative correlation with access to safe use information, perception of the effectiveness of pesticides, wearing of personal protection equipment, and reading the instructions on pesticide labels before use (Table 8).

**Table 8:** Correlation test between the increased use of pesticides and demographic variables.

Variable	Pearson Correlation	p-value
Gender of respondent	−0.119	0.055
The age category of the respondent	−0.114	0.065
The highest education level attained	−0.009	0.886
Access to safe use information	−0.142^a^	0.022
Perception of the effectiveness of pesticides	−0.143^a^	0.022
Mix more than one pesticide during spraying	0.225^b^	0.000
Number of crops grown consecutively	0.264^b^	0.000
Do you wear personal protective equipment	−0.258^b^	0.000
Frequency of pesticide spraying	−0.001	0.982
Region of respondent	0.553	0.000
Read instructions on pesticide labels before use	−0.183^b^	0.003

a . Correlation is significant at the 0.05 level (2-tailed).

b . Correlation is significant at the 0.01 level (2-tailed).

Determinants of farmers’ increased use of pesticides are presented in Table 9 from the results of the probit regression model. The predictors tested were: region of the farmer, gender, age, education level, number of vegetable crops each farmer grows, farm size, perception of the effectiveness of pesticides, mixing practices, frequency of pesticide application, access to information on pesticide use, source of pesticides, use of personal protection equipment and the tendency of farmers to read pesticide label. The choice of explanatory variables in the probit model was based on the widely reported factors and risk behaviors in pesticide handling [5,16,25,[37], [38], [39]]. Among the variables tested, the region of the farmer, the number of vegetable crops grown, and the mixing of pesticides positively contributed to the likelihood of increased use of high levels of pesticides.

**Table 9:** Determinants of farmers’ increased use (Results of the Binary Probit Model).

Explanatory variables	Estimate	Std. Error	Sig.	Wald Chi-Square	95 % Wald Confidence Interval	
					Lower	Upper
Region	1.488	0.2236	0.000	44.273	1.049	1.926
Gender	0.65	0.2921	0.126	4.957	0.078	1.223
Age category	0.254	0.162	0.117	2.451	−0.064	0.571
Highest level of education	0.107	0.2593	0.68	0.17	−0.401	0.615
Number of crops grown	0.147	0.0468	0.002	9.919	0.056	0.239
Farm size	−0.395	0.2077	0.057	3.616	−0.802	0.012
Perception of the effectiveness of pesticides	−0.555	0.2429	0.022	5.221	−1.031	−0.079
Mixing of pesticides	0.592		0.012	6.293	0.129	1.054
Access to safe use information	−0.717	0.4284	0.04	4.205	−1.718	−0.039
Frequency of pesticide spray	−0.2	0.0932	0.116	2.47	−0.329	0.036
Source of pesticides	−0.07	0.3145	0.823	0.05	−0.687	0.546
Wearing of PPEs	−0.349	0.1698	0.04	4.222	−0.682	−0.016
Reading of Pesticides Label	−0.446	0.2136	0.037	4.351	0.027	0.864

On the contrary, farmers' perception of the low effectiveness of insecticides, access to information on pesticide-safe use, use of PPEs, and reading pesticide labels negatively influenced the excessive use of pesticides. In other words, the perception of the low effectiveness of pesticides, limited access to information on the safe use of pesticides, and low use of protective gear among farmers increased the likelihood of farmers using pesticides indiscriminately. Other variables including gender, age category, level of education, farm size, frequency of pesticide spray, and source of pesticides showed no significant contribution in influencing farmers’ decision to excessively apply high levels of pesticides.

## 4 Discussion

Smallholder vegetable production systems were informal and uncontrolled, with farmers generally lacking insect pest control knowledge and education on the safe use of pesticides. The farming population involved was usually young with a low level of education. Insecticides followed by fungicides and herbicides were the main pesticides used. These pesticides had been previously reported in Tanzania [2,40] and elsewhere [3,41,42]. Excessive pesticide use that ultimately increases exposure risk can therefore be explained by inefficient pesticide control at the farm level.

The levels of pesticide use per acre of cropping land in smallholder vegetable production were considerably high with increased frequency application, unlike low levels of pesticide use reported in rice production [43]. The reported dose rates were far above the world average of 1.2–1.8 kg a.i/ha pesticide use per land [44]. However, these levels were below the top world pesticides users per cropping land such as the Republic of Korea (10 kg a.i/ha), Belize (11 kg a.i/ha), Netherlands (11 kg a.i/ha), Seychelles (12 kg a.i/ha), Japan (12 kg a.i/ha), Ecuador (14 kg a.i/ha), Israel (15 kg a.i/ha), Oman (16 kg a.i/ha), Maldives (17 kg a.i/ha) and Lucia (20 kg/ha) [44]. Owing to the high application rates and pesticide doses, high levels of pesticide residues are presumed to be found in the environment thereby increasing the exposure risks. This is because approximately 90 % of agricultural pesticide applications never reach their target organisms but are, instead, dispersed through the air, soil, and water [45] which results in the accumulation of their residues and metabolites in soil at unacceptably high levels.

Pesticides active ingredient per hectare found in this study were also high as compared to other countries in Africa, even far above the projected 2040 baseline level of pesticide use of 0.14 kg/ha in East Africa and 0.32 kg/ha in Southern Africa [46]. In Zimbabwe the use insecticides [47] had been reported to range from 0.20 to 0.67 kgs per hectare per application, while vegetable farmers in Ghana [48] reported rates varying from 0.02 to 1 kgs per hectare per application. Furthermore, high spraying frequencies averaging once every week throughout the farming season was evident. Similar practices of overuse of pesticides by farmers have been reported in previous studies [3,29,49]. This practice result in a high accumulation of pesticide residues in the environment and food produce [50,51], affecting the biological diversity of the ecosystem. Limited knowledge of pesticide use among farmers is assumed to be the reason for the non-use of the recommended amounts of pesticides [14].

Pesticide's use had increased tremendously. The study identified over 75 different pesticides being used by farmers, as opposed to 40 different types reported previously in vegetable farming from Tanzania [17]. However, this trend was accompanied by a changing trend of applying mixed pesticide formulations. An increasing trend in pesticide use had been previously reported [14]. The gradual change in the application of mixed active ingredients might be due to increased insect pest resistance to individual organophosphates, carbamates, and pyrethroids, which have been traditionally used [5]. Excessive pesticide use has a critical implication on the health of farmers, consumers of vegetable crops, and the environment [2,8,52,53].

Generally, farmers’ awareness of the disposal and management of pesticides was low. Most empty pesticide containers were left in the field during the whole farming season and collected/burnt during field preparation in the next farming season. The tendency to leave pesticide leftovers and wastes on the farm or dumping directly onto the land or into water [30] increases continuous exposure risks to both humans and wildlife [54]. Similar disposal methods and farmers reusing empty pesticide containers for domestic purposes have been previously reported [5,49,55,56]. Burning empty containers directly without triple rinsing produces toxic fumes which increase the risk of respiratory health problems while burying increases the risks of contaminating both surface and underground water and adverse effects on non-target organisms including aquatic and terrestrial ecosystems [57].

The use of highly toxic pesticides increases exposure risks for farmers. Organophosphate and carbamate pesticides prevailed in smallholder vegetable production, which poses a remarkable health concern due to pesticides' neurotoxic effects due to the depression of acetylcholinesterase enzyme activities [4]. These pesticides have been reported to dominate agricultural production in developing countries [3,8,28]. The reported prevalence of WHO Class II pesticides also poses high health risks. Profenofos, chlorpyrifos, and cypermethrin among pesticides commonly used have been associated with decreased ecological functioning, degradation of natural vegetation, decreased number of biological species, and the depletion of fish resources [58].

The increased pesticides had been associated with various factors including the region of the farmers, the number of vegetable crops grown, and the mixing of pesticides. In regions with the predominant use of pesticides, farmers are persuaded to use more pesticides. Similar influence of geographical location on pesticide handling practices has been reported among vegetable farmers in Kenya [56]. Farmers' pesticide handling is, therefore, learned over experience [3], which ultimately influences farmers' behavior and develops into a common practice and lifestyle resulting in social patterned pesticide-handling practices [49], which is translated into the belief that the more pesticides the farmer uses, the more progressive the farmer is. Most farmers cultivating more than one vegetable crop consecutively has also been found to influence farmers’ likelihood of using more pesticides. This is fueled by the growing demand for increased productivity in a limited area of land. Extensive use of pesticides also results in the development and evolution of insect pest resistance to pesticides [59].

Mixing of pesticides during spraying was also a determinant for increased pesticide use. Farmers mixing more than one pesticide had been previously reported [3]. Mixing more than one pesticide may increase toxicity of pesticides due to the additive toxicity of the resultant formulations as well as in chemical interactions among pesticide molecules, resulting in more severe effects on both farmers and consumers [60]. This practice may also result in chemical reactions of pesticide components that may change the potential of active ingredients, hence, lowering the effectiveness of the pesticides due to antagonistic effects. This may explain the considerably high proportion of farmers reporting lower effectiveness of pesticides in the control of insect pests. Farmers mix pesticides contrary to the instructions on pesticide labels [61], mixing done mainly in tanks which fosters farmers' direct contact with pesticides [62]. Such practices subject the farming population to the development of numerous diseases including non-Hodgkin's lymphoma, multiple myeloma, pancreatic, stomach, liver, bladder, and gall bladder cancer, prostate cancer, Parkinson's disease, and reproductive outcomes [63,64]. Farmers are, therefore, at high risk of exposure during pesticide application due to poor monitoring and a lack of well-trained experts to train and guide them on the safe use of pesticides [49,65]. These health risks depend not only on how toxic the pesticide is but also on the level of exposure [33]. Poor pesticide handling and haphazard pesticide spraying have been associated with contamination of food produced with hazardous chemicals from pesticides [[66], [67], [68]].

Farmers' perception of the low effectiveness of pesticides, lack of access to information on safe pesticide use, poor use of safety gear, and inability to read pesticide labels influenced the level of pesticide use. Farmers' perception of the low effectiveness of pesticides has been reported to drive the use of high pesticide concentrations [3,29]. Very few farmers received pesticide-safe use information mainly from pesticide retailers. Similar findings had been reported among farmers in Pakistan, where only 12.9 % had access to information about pesticide use [3]. Lack of access to safe use information negatively affects farmers' attitudes resorting to increased use of pesticides. A study on farmers’ training on pesticide use and safety behavior among rural farmers in northern Greece revealed that most trained farmers showed higher levels of knowledge of pesticide use, pesticide hazard control, and safety behavior than non-trained farmers [39]. Since knowledge of pesticide risks had been found to increase with formal education and training on pesticide-safe use [14]. Farmers receiving information from pesticide retailers was also reported among tobacco farmers in Greece [69]. Previous studies have shown that receiving advice on pesticide use from pesticide retailers increases inappropriate handling practices of pesticides significantly [56]. Negative effects of pesticides resulting from poor use of pesticides can be minimized by educating and training farmers on the judicious use of pesticides [14,70]. These findings complement what had been reported earlier that pesticide handling is worse in developing and under-developed countries because safety and regulatory guidelines on pesticide handling are hardly practiced [11] and most farmers in these countries are not adequately informed about the hazards associated with the chemicals [17,37]. Poor use and handling practices observed in this study may be a result of poor information on pesticide use provided by pesticide retailers, lack of training on insect pest control and safe use of pesticides as well a low level of formal education among farmers. Improved knowledge of pesticide use can ultimately avert human and environmental pesticide exposure by minimizing their use and replacing highly toxic pesticides with those of low toxicity [71].

Personal protection equipment (PPE) was not effectively used when handling pesticides. The use of gloves and boots, which is the minimum PPE for most pesticide products [26] was not a common practice. Similar findings were observed among farmers in Pakistan where individuals often did not use PPE when working with pesticides [14,38,52]. In this study, wearing PPE was found to influence negatively the use patterns of pesticides. Farmers who did not use PPE were more likely to excessively use pesticides. Those wearing PPE might be more conscious of the safe use, hence, precautionary using low levels of pesticides. Poor use of PPE and high application frequencies imply increased farmers' direct contact with pesticides, potentially exposing them to pesticides during the preparation and application and the cleaning-up of spraying equipment [26]. This also translates into an increased risk of exposure because a large proportion of pesticide absorbed into the body comes from dermal exposure [72]. Pieces of evidence of pesticide intoxication exerting the strongest positive influence on PPE use have been reported [38], highlighting the importance of PPE use in reducing pesticide exposure among farmers. Increasing farmers’ awareness of the proper use of PPE would also result in the judicial use of pesticides.

Reading pesticide labels negatively contributed to the increased use of pesticides. Similar findings had been reported among Greek tobacco farmers where only a few farmers relied on the information found on the product label, implying that essential information about pesticide handling and safety issues found on pesticide labels are not effectively communicated to farmers [69]. As a result, farmers who did not read instructions on pesticide labels were more likely to excessively use high levels of pesticides. In situations of farmers with low levels of education, reading the instructions written on the pesticide containers had been reported as not very effective [14]. Nonetheless, pesticide product labels are critically important for consumers to improve safely and legally the use of pesticide products. To minimize pesticide exposure and promote pesticide-safe use, improved pesticide product labels with recommended safe durations have to be included on each pesticide product [73].

The use of pesticides registered for other crops as well as in the treatment of ectoparasites and ornamental crops indicates a knowledge gap in the utilization of information provided on the pesticide label. Studies have reported that farmers could use any pesticide product to control pesticides. Their desire to eliminate crop pests drives them to use any pesticide, unregistered, or banned pesticide products [27,74]. Overall, the results attained in this study support the hypothesis that in reducing the effects of insect pests, farmers have resorted to using more pesticides with increased amounts and concentrations of pesticides, using toxic pesticides (pesticides belonging to high-toxicity categories), and providing farmers with good knowledge of pesticide use can substantially reduce human and environmental pesticide exposure.

## 5 Conclusion

Pesticides will continue to be an essential component of agricultural production in farmers' efforts to control insect pests and increase farm income. In this study, determinants of increased pesticide use and drivers of farmers' behavior in changing pesticide use practices were assessed. The novelty of this study emanates from the quantification of pesticide active ingredients contained in pesticide mixtures and the causal link of increased pesticide use with farmers’ risk behavior, production typologies, and pesticide handling practices in smallholder horticultural production,

High quantities of pesticides excessively used by smallholder vegetable producers exceed world averages, showing that smallholder horticultural producer applies high levels of pesticides per cropland exceeding both African and world averages. Pesticide use had almost doubled during the last decade with farmers generally lacking insect pest control knowledge and education on the safe use of pesticides. Furthermore, there is ineffective control and monitoring of pesticides in smallholder vegetable production systems. Increased use of a combination of different pesticide formulations, mixing different pesticides, and increased volumes of pesticides sprayed on a single spray per acre indicate high levels of pesticide residues in vegetable produce and the environment. Hence farmers and the general population are at risk of a wide range of detrimental health effects and pesticide-related pathologies due to occupational and environmental exposure. The fate of pesticide use in smallholder vegetable production systems is therefore the culmination of serious health and environmental implications. Excessive pesticide use in controlling crop insect pests and diseases, escalated by an increased number of crops, improper use of PPE, and mixing practices of different pesticides subject the general population to pesticide environmental exposure thereby jeopardizing the sustainability of smallholder vegetable production in Tanzania. Low-level knowledge, high frequency of pesticide use, and mixing practices imply that both human and environmental exposure to pesticides is a serious matter of concern.

Changing pesticide handling practices necessitates a review of the current registration process of pesticides by considering the use of greener pesticides as well as the reconsideration of the newer formulations that are safer and can replace the more toxic pesticides. Poor pesticide use can be minimized through effective pesticide monitoring strategy at the farm level, specialized training on behavioral change with emphasis on the reduction of pesticide use per acre of production land and integrated pest management strategies. Regular training for extension officers on current and emerging issues of insect pest control and safe use of pesticides is vital. Insect pest resistance to individual pesticide formulation needs to be scientifically established, and the reason for pesticide mixing should be justified scientifically. Environmental exposure studies are also needed to establish the ecological effects of the changing patterns of pesticide use.

## Data and code availability statement

Data included in article/supplementary material is referenced in the article.

## Ethics statement

Review and/or approval by an ethics committee was not needed for this study because it was undertaken as part of surveillance and pesticide monitoring activities which is a legal mandate of the lead institution, the Tanzania Plant Health and Pesticides Authority (TPHPA)

## Declaration of competing interest

The author declares the following financial interests/personal relationships which may be considered as potential competing interests:Dr. Jones Ackson Kapeleka reports that the Tanzania Horticultural Association (TAHA) provided financial support. However, the author reports no relationship with the association and therefore declares that he has no known competing financial interests or personal relationships that could have appeared to influence the work reported in this paper.
