# Supplementary material for: Quantification of pesticide dosage and determinants of excessive pesticide use in smallholder vegetable production systems in Tanzania
Source: Heliyon. 2024 Dec 7;10(24):e41070. doi: 10.1016/j.heliyon.2024.e41070 (PMC11665471; doi:10.1016/j.heliyon.2024.e41070)
Supplement: Multimedia component 1 [file mmc1.pdf]

APPENDIX 1 QUESTIONAIRE

ASSESSMENT OF PESTICIDES USE DYNAMICS AND PRACTICES AMONG SMALLHOLDER VEGETABLE PRODUCERS IN TANZANIA

1.0 Personal information

Region............................. District...

Ward.. Village. ....

GPS Coordinates:

Please circle in the corresponding answer accordingly

1.1 Sex of the respondent [1] Male [2] Female

1.2 Which age category are you? -------------
   [1] 15-24 years
   [2] 25-34 years
   [3] 35-44 years
   [4] 45-54 years
   [5] 55-64 years
   [6] 65 years and above

1.3 What is the highest level of education have you attained?
   [1] Not gone to school
   [2] Class seven
   [3] Form IV
   [4] Form VI
   [5] Certificate or Diploma
   [7] University Degree

1.4 Which crops do you grow? (Multiple answers allowed)
   [1] Tomatoes
   [2] Onions
   [3] Sweet paper
   [4] Watermelons
   [5] Amaranths
   [6] Night shade
   [7] Chinese cabbage
   [8] Kale
   [9] Carrots
   [10] Cabbage
   [11] Cucumbers
   [12] African eggs plant
   [13] Okra
   [14] Others (Specify)…………………...

1.5 Specify the size of your field in acres.........

2.0 Agricultural production and pests/disease situation

2.1 What is the major purpose of agriculture production you are doing:

[1] Home consumption only

[2] Home consumption and business

[3] Business only.

2.2 What pests normally affects your field

[1] Insect Pests

[2] Birds

[3] Plant diseases

[4] Wildlife

[5] Thieves

[6] Fungal diseases

[7] Mouse

[8] Other (Specify)...

2.3 List the pests/diseases that appear each season in your field for each of the crops?

CROP	PESTS/DISEASES			
Tomatoes				
Onions				
Watermelons				
Sweet paper				
Night shade				
Chinese cabbage				
Kale				
Cucumber				
Cabbage				
Carrota				
Amaranths				
African egg plant				
Okra				

2.4 Have you ever met agricultural experts for advice on how to control crop pests?

[1] Yes [2] No

3.0 Use of pesticides in agriculture in horticultural production

3.1 Have you ever received advice / training on effective use of pesticides in controlling disorders?

[1] Yes [2] No (Go to question 3.3)

3.2 If yes, where did you get the advice/training?

[1] Extension officer

[2] Researchers

[3] Pesticides Companies/sellers

[4] Non-Governmental Organizations

[5] Government institutions.. ......

3.3 What exactly leads you to or even pushes you to spray the pesticides in the field?

[1] The presence of insects in the field

[2] When I see my neighbor/friend spraying
[3] Advice from the extension officer
[4] Advice from pesticides retailers
[5] Other reasons ....

3.4 Do the pesticides used satisfy you needs?
[1] Yes [2] No

3.5 If not, specify the limitations you see?
....

3.6 How do you perceive current effectiveness of pesticides compared to the last two years?
[1] Has increased [2] It has remained the same [3] decreased [4] I do not know

3.7 Do you mix more than one type of pesticides when spraying your crop?
[1] Yes [2] No

3.8 If yes why do you mix pesticides?
....
....

3.9 Where do you mix your pesticides?
[1] Farm [2] Pesticides store [3] Other place (specify) -------------------------------

3.10 How do you describe your pesticides spraying schedule for the day?
[1] From morning till evening no resting
[2] From morning till 12 noon, then 4pm until 6:00 pm
[3] From morning till finish mixed pesticides
[4] Other (Explain briefly) ....

3.11 Specify the pesticides used in each crop you grow

**CROP**	**PESTICIDES USED** *Eg: 1. Agrocron 720 EC, 2. Snow plus 550EC, 3. Wilthane 72WP, 4. Selecron 720EC, 5. Prosper 720EC*				
Tomatoes	1.	2.	3.	4.	5.
Onions	1.	2.	3.	4.	5.
Watermelons	1.	2.	3.	4.	5.
Sweet paper	1.	2.	3.	4.	5.
Night shade	1.	2.	3.	4.	5.
Chinese cabbage	1.	2.	3.	4.	5.
Kale	1.	2.	3.	4.	5.
Cucumber	1.	2.	3.	4.	5.
Cabbage	1.	2.	3.	4.	5.
Carrota	1.	2.	3.	4.	5.
Amaranths	1.	2.	3.	4.	5.
African egg plant	1.	2.	3.	4.	5.
Okra	1.	2.	3.	4.	5.
	1.	2.	3.	4.	5.

3.12 What device do you use during mixing of pesticides?

[1] Barrel (liter ......)
[2] Knapsack lit.........
[3] Bucket lit.............
[4] Other tool (Specify name and volume in liters) ....

3.13 What quantity (liter / kg) of any pesticides that you mix in the container mentioned in question 3.12 above.

ZAO	**QUANTITY OF PESTICIDES MIXED** (liter / kg) per Unit production area ***Example: 1. Agrocron 720 EC (2 l) 2. Snow plus 550EC (cc 25) 3. Wilthane 72WP (1 Kg) 4. Selecron 720EC (3 l) 5. Prosper 720EC (1.5 l)***				
Tomatoes	1.	2.	3.	4.	5.
Onions	1.	2.	3.	4.	5.
Watermelons	1.	2.	3.	4.	5.
Sweet paper	1.	2.	3.	4.	5.
Night shade	1.	2.	3.	4.	5.
Chinese cabbage	1.	2.	3.	4.	5.
Kale	1.	2.	3.	4.	5.
Cucumber	1.	2.	3.	4.	5.
Cabbage	1.	2.	3.	4.	5.
Carrota	1.	2.	3.	4.	5.
Amaranths	1.	2.	3.	4.	5.
African egg plant	1.	2.	3.	4.	5.
Okra	1.	2.	3.	4.	5.

3.14 Mention the number of drums (volume in the barrel) of pesticides that are used in the field at one moment

**CROP**	**Size the farm in acres**	**Number of drums/day**	**No. of working hours/day)**	**No. of spraymen/day**	**Total No. of working days**
Tomatoes					
Onions					
Watermelons					
Sweet paper					
Night shade					
Chinese cabbage					
Kale					
Cucumber					
Cabbage					
Carrota					
Amaranths					
African egg plant					
Okra					

4.0 Safety precautions during pesticides applications in the field

4.1 Do you read labels before using pesticides?
[1] Yes [2] No

4.2 If Yes, do you follow the instruction given?
[1] Yes [2] No

4.3 What do you do with empty pesticides containers?
[1] Fetching drinking water
[2] Buy food
[3] Food storage
[4] thrown in dustbin
[5] Burned
[6] Buried under the ground
[7] Other applications (specify) ....

4.4 On average, how often do you use (spray) pesticides?
[1] Once a week [2] Twice a week [3] Three times a week [4] Once in two week [5] Once a month [6] Other (Explain) ....

4.5 What is the pre-harvesting period after spraying? ……………days

4.6 Where do you get pesticide?
[1] Pesticide’s retail shops [2] Open / auction market [3] Wholesale pesticides shops

4.7 Do you wear protective equipment when dealing with pesticides?
[1] Always [2] rarely [3] No

4.8 Where do you store pesticides?
[1] Pesticide’s store [2] Kitchen [3] Bathroom/toilet [4] General Stores [6] Ceiling board [7]

4.9 Describe the things you do while dealing with pesticides
[1] Eat 1. Yes 2. No
[2] Drinking 1. Yes 2. No
[3] Smoking 1. Yes 2. No

4.10 Specify the protective equipment you wear when dealing with pesticides (tick where appropriate)

**Protective Equipment**	**No**	**Have but don’t use**	**Use but worn-out**	**Use and in good condition**
Gloves				
Boots				
Respirator				
Mask				
Goggles				
Overall				
Head cover				
